# Veterinary Medical Association of New York County

**Published:** 1898-01

**Authors:** Robert W. Ellis

**Affiliations:** Secretary


### SOCIETY PROCEEDINGS.

### VETERINARY MEDICAL ASSOCIATION OF NEW YORK COUNTY.

The Association was called to order at 8.30 o'clock, on Wednesday, December 1, 1897, at the New York Academy of Medicine, by the President, Dr. Huidekoper. The following members responded to roll-call: Bretherton, C. C. Cattanach, J. S. Cattanach, Delaney, Ellis, Farley, Huidekoper, Hanson, MacKellar, Neher, O'Shea, Robertson, and Ryder—13.

The committee appointed by the President to search the register in reference to the qualification to practice in New York County of Messrs. Grenside and Hingston, not being present, President Huidekoper reported that Hingston was not eligible to qualify by virtue of the penal code, he having been guilty of a felony.

The following communication from Dr. John J. Cattanach was next read by the Secretary:

Dr. R. W. Ellis

*Dear Sir:* As I am about to retire from the profession, would beg of you to hand in my resignation to the society.

Very truly yours,

John J. Cattanach.

Moved and seconded that the resignation be accepted with the regrets of the Association; carried.

Next in order was the election of officers for 1898. Nominations were as follows:

For President, Dr. Huidekoper and Dr. Bell; moved and seconded, nominations close; carried. Vice-President, Dr. Robertson, Dr. Hanson; moved and seconded, nominations close; carried. Treasurer, Dr. C. C. Cattanach, Dr. Ryder, and Dr. Hanson; moved and seconded, nominations close; carried. Secretary, Dr. Ellis; moved and seconded, nominations close; carried.

Result by ballot for first three offices: Dr. Huidekoper was elected President; Dr. Robertson, Vice-President, and Dr. Hanson, Treasurer. In election of Secretary, it was moved and seconded that the By-laws be suspended and the election declared unanimous; carried.

Next business in order was the Treasurer's report. The retiring Treasurer, Dr. C. C. Cattanach, submitted the following report for the two years during which he had served the Association in that office:

| | | |
|---|---|---|
| February, 1896, balance received from former Treasurer, | $13 18 | |
| Received from Secretary p. . . . . . . | 534 00 | |
| | | $547 18 |
| Expenditures amounting to . . . . . . | | 524 90 |
| Leaving a balance in the treasury of . . . . . | | $22 28 |

Appended to this was an itemized list of the expenditures.

Moved and seconded, that the report be accepted; carried.

Dr. Neher recalled a case reported by him some time ago, which he had diagnosed as purpura hemorrhagica, which he said was entirely wrong, as he has since had evidence that the conditions found were due to traumatism. Remarks by members.

Moved and seconded that the meeting adjourn; carried.

ROBERT W. ELLIS, D.V.S.,
Secretary.

